# Segmentation of Coronary Calcified Plaque in Intravascular OCT Images Using a Two-Step Deep Learning Approach

**DOI:** 10.1109/access.2020.3045285

**Published:** 2020-12-16

**Authors:** JUHWAN LEE, YAZAN GHARAIBEH, CHAITANYA KOLLURU, VLADISLAV N. ZIMIN, LUIS AUGUSTO PALMA DALLAN, JUSTIN NAMUK KIM, HIRAM G. BEZERRA, DAVID L. WILSON

**Affiliations:** 1 Department of Biomedical Engineering, Case Western Reserve University, Cleveland, OH 44106, USA; 2 Cardiovascular Imaging Core Laboratory, Harrington Heart and Vascular Institute, University Hospitals Cleveland Medical Center, Cleveland, OH 44106, USA; 3 Interventional Cardiology Center, Heart and Vascular Institute, University of South Florida, Tampa, FL 33606, USA; 4 Department of Radiology, Case Western Reserve University, Cleveland, OH 44106, USA

**Keywords:** Intravascular optical coherence tomography, coronary calcified plaque, major calcification, two-step deep learning

## Abstract

We developed a fully automated, two-step deep learning approach for characterizing coronary calcified plaque in intravascular optical coherence tomography (IVOCT) images. First, major calcification lesions were detected from an entire pullback using a 3D convolutional neural network (CNN). Second, a SegNet deep learning model with the Tversky loss function was used to segment calcified plaques in the major calcification lesions. The fully connected conditional random field and the frame interpolation of the missing calcification frames were used to reduce classification errors. We trained/tested the networks on a large dataset comprising 8,231 clinical images from 68 patients with 68 vessels and 4,320 *ex vivo* cadaveric images from 4 hearts with 4 vessels. The 3D CNN model detected major calcifications with high sensitivity (97.7%), specificity (87.7%), and F1 score (0.922). Compared to the standard one-step approach, our two-step deep learning approach significantly improved sensitivity (from 77.5% to 86.2%), precision (from 73.5% to 75.8%), and F1 score (from 0.749 to 0.781). We investigated segmentation performance for varying numbers of training samples; at least 3,900 images were required to obtain stable segmentation results. We also found very small differences in calcification attributes (e.g., angle, thickness, and depth) and identical calcium scores on repetitive pullbacks, indicating excellent reproducibility. Applied to new clinical pullbacks, our method has implications for real-time treatment planning and imaging research.

## I. INTRODUCTION

Coronary calcified plaque (CCP) is an important marker of early atherosclerosis. CCP is highly prevalent in patients with coronary heart disease and leads to reduced vascular compliance, abnormal vasomotor responses, and impaired myocardial perfusion [1], [2]. The severity of CCP is strongly associated with the degree of atherosclerosis, and the extent of CCP distribution is linked to higher rates of complications and worse outcomes during or after percutaneous coronary intervention (PCI), the most widely performed intervention for coronary heart disease [3]. Accurate identification and quantification of CCP can help guide the PCI treatment plan and determine efficacy to improve patient outcomes.

Intravascular optical coherence tomography (IVOCT) is a high contrast, high-resolution imaging modality that produces cross-sectional images of coronary arteries using a near-infrared light. Compared to intravascular ultrasound (IVUS), IVOCT provides better penetration depth for detection of calcifications and relatively high axial (12–18 *μm* vs. 150–250 *μm* from IVUS) and lateral (20–90 *μm* vs. 150–300 *μm* from IVUS) resolution [4]. IVOCT is a promising modality for quantifying calcifications in the inner layers of the coronary vessels and for identifying thin cap fibroatheroma, plaques that are vulnerable to rupture [5]. Additionally, this modality is uniquely capable of guiding PCI and assessing intervention outcomes [6]. IVOCT-guided PCI may bring additional value to patient treatment when compared to PCI-guided X-ray angiography alone [7]. These advantages have led to greater application of IVOCT for both clinical and research purposes. Despite its advantages for imaging intravascular plaque, IVOCT limitations include the need for specialized training and the lack of fully automated plaque characterization. Each IVOCT pullback generally includes 300–500 image frames depending on the settings. Complete manual annotation of coronary plaques for research requires careful consideration of image characteristics, which is time consuming, labor intensive, and subject to high inter- and intra-observer variability [8]. In addition, manual annotation is impossible for real-time treatment planning. Thus, there is a clear need for automated plaque analysis in IVOCT images.

Previous studies have used machine/deep learning to identify the plaque components in IVOCT images. Ughi *et al.* [9] proposed the systematic characterization of atherosclerotic tissues using textural features combined with the optical attenuation coefficient. Using random forest, the overall classification accuracy was 81.5%. Rico-Jimenez *et al.* [10] extracted profile morphological features and classified them as either intimal-thickening, fibrous, fibro-lipid, or superficial-lipid, based on a linear discriminant analysis algorithm. Prabhu *et al.* [11] developed a machine learning approach to identify fibrolipidic and fibrocalcific plaques using a comprehensive set of hand-crafted features. Some studies have suggested that the optical attenuation coefficient of each plaque is a good indicator for discriminating plaque contents [9], [12]–[14]. Several deep learning algorithms (e.g., U-net [15], SegNet [16], and Deeplab v3+ [17]) have been applied to IVOCT image analyses. Yong *et al.* [18] proposed a linear-regression convolutional neural network (CNN) to segment the lumen border and obtained the mean absolute error of 21.9 microns. Kolluru *et al.* [19] used the CNN model comprising two convolutional and two max-pooling layers for A-line classification in IVOCT images. The F1 scores for fibrolipidic and fibrocalcific classes were 0.72 and 0.77, respectively. Gessert *et al.* [20] employed two pre-trained deep learning networks, ResNet 50-V2 [21] and DenseNet121 [22], for a frame-wise plaque identification. They obtained an accuracy of 91.7%, sensitivity of 90.9%, and specificity of 92.4%. Zhang *et al.* [23] segmented the atherosclerotic plaques in IVOCT images using the CNN and random walk algorithm. Compared to the manual labeling, the Jaccard similarity coefficients of fibrotic and calcified plaques were 0.876 and 0.864, respectively. Abdolmanafi *et al.* [24] proposed a VGG-based fully convolutional network to characterize four different lesion types-calcification, fibrosis, macrophage, and neovascularization. They achieved an approximate overall accuracy up to 90% for all lesion types. In a previous report, we provided both pixel-wise and A-line-based classifications using fully automated deep learning models [25]. We found that SegNet significantly improved sensitivities compared to the Deeplab v3+ network. A few groups have combined machine and deep learning models. Abdolmanafi *et al.* [26], [27] used pre-trained deep learning networks as feature extractors and identified the lumen border and plaques (e.g., calcification, fibrosis, normal intima, macrophage, media, and neovascularization). Most recently, our group combined previously reported lumen morphology features [11] with deep learning features [19] and classified each A-line as either fibrolipidic or fibrocalcific plaques [28].

Previous studies have shown that a two-step approach provides better classification/segmentation as compared to a one-step approach. Wang *et al.* [29] proposed a two-step CNN method consisting of a selection-CNN, which identified a region of interest, and a segmentation-CNN for automatically segmenting adipose tissue in computed tomography images. Results showed that the two-step approach provided significantly better segmentation performance than the one-step approach. Eftekhari *et al.* [30] proposed a two-step CNN method to detect the microaneurysm in fundus images. They found that the two-step approach not only corrects for the imbalanced dataset problem, but also reduces training time. Hong *et al.* [31] also promoted a two-step deep neural network for segmentation of deep white matter hyperintensities (WMHs) in magnetic resonance imaging data. For real-world data, Kong *et al.* [32] designed an architecture combining both attention and local reconfigurations to gather task-oriented features and achieved significant improvement compared with the one-step method. In each IVOCT image pullback, we obtain as many as 540 image frames, with a small fraction containing calcifications. In addition, there is significant variability among calcifications, more so than might be found in organ segmentation, for example. As a result, we hypothesized that a two-step approach may be more effective, in which the network is only required to learn the calcification segmentation task in the second semantic segmentation step, after the identification step, thereby avoiding the need to learn the variability in the larger set of image frames. In addition, if a reliable first step identifies frames with calcifications, this would enable a great reduction in manual labeling effort and would reduce the number of frames required to train the segmentation network.

In this paper, we built on our previous studies to test a fully automated calcium segmentation method using a two-step deep learning approach to analyze IVOCT images. The proposed method first localizes the major calcification lesions using the 3D CNN model. A deep convolutional encoder-decoder architecture (SegNet [16]) subsequently provides pixel-wise classifications of calcified plaques. A fully connected conditional random field (CRF) is applied to standardize classification over larger regions. Classification results are shown based on the probability of each pixel. To avoid potential data distortion, all image processing steps are performed in the raw IVOCT image in (*r*, *θ*) domain. We examined the accuracy of this new approach in a large set of IVOCT images, as well as robustness and reproducibility.

This study has several important contributions. First, by using a two-step deep learning approach, we can successfully process entire pullbacks, eliminating the need for a physician to focus the attention of a deep learning solution to a particular lesion. Second, our approach provides improved segmentation results with scarce data and greatly reduces the manual labeling efforts. Third, we evaluate our method in various ways (e.g., different pre-trained networks and loss functions) to optimize the segmentation performance. Fourth, the appropriateness of the sample size for network training is investigated. We use a rational approach to determine whether the training sample size is sufficient. Fifth, we test the reproducibility of the proposed method on ex vivo data set.

## II. IMAGE ANALYSIS METHODS

### A. PRE-PROCESSING

**FIGURE 1.**
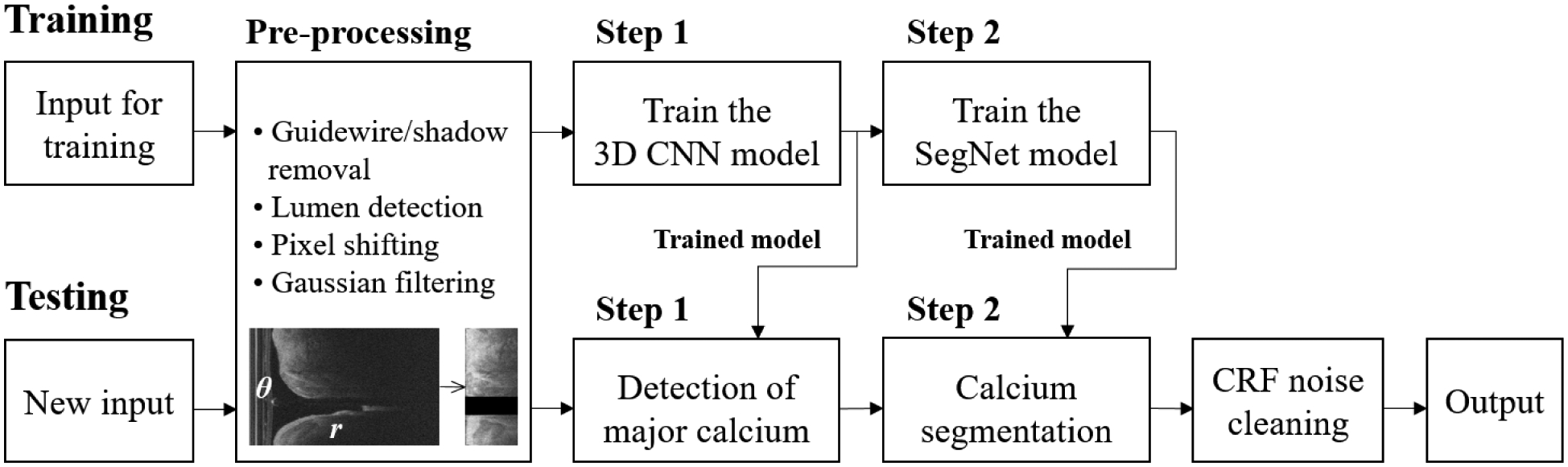
Overall workflow of the proposed two-step deep learning approach for calcium segmentation. Pre-processing is applied to the raw IVOCT image in (*r*, *θ*) domain. After pre-processing, the size of the input image is reduced from 968 × 448 to 200 × 448 without any data loss. The trained 3D CNN (step 1) was used to determine the major calcification lesions from the entire pullback. The calcified plaques were segmented using the trained SegNet model (step 2). Classification noises were reduced using a fully connected CRF method. The output label was transformed back to the original size from 200 × 448 to 968 × 448.

In our previous study, we found that some pre-processing steps significantly improved segmentation performance in IVOCT images [25]. In this study, pre-processing was fully automatically applied in the polar (*r*, *θ*) domain raw IVOCT images. First, the guidewire and corresponding shadow region were detected using dynamic programming [33]. Briefly, when the first boundary (upper or lower) of the guidewire was identified, the small search mask was utilized to locate the second boundary (lower or upper). Then, the guidewire and corresponding shadow regions were removed. Second, the lumen boundary was detected using the semantic segmentation method using deep learning [34]. Third, to help align the tissues, each A-line of the resulting image was pixel-shifted to the left allowing all A-lines to have the same starting pixel along the radial direction. Fourth, we set a certain range (1 *mm*, 200 pixels) in the *r* direction as the region of interest (ROI), since IVOCT has limited penetration depth. Finally, Gaussian filtering was applied to reduce speckle noise with a standard deviation of 1 and filter size of (7,7). Fig. 1 shows the overall workflow of the two-step deep learning approach.

### B. DETERMINATION OF THE MAJOR CALCIFICATION LESIONS USING 3D CNN (STEP 1)

**FIGURE 2.**
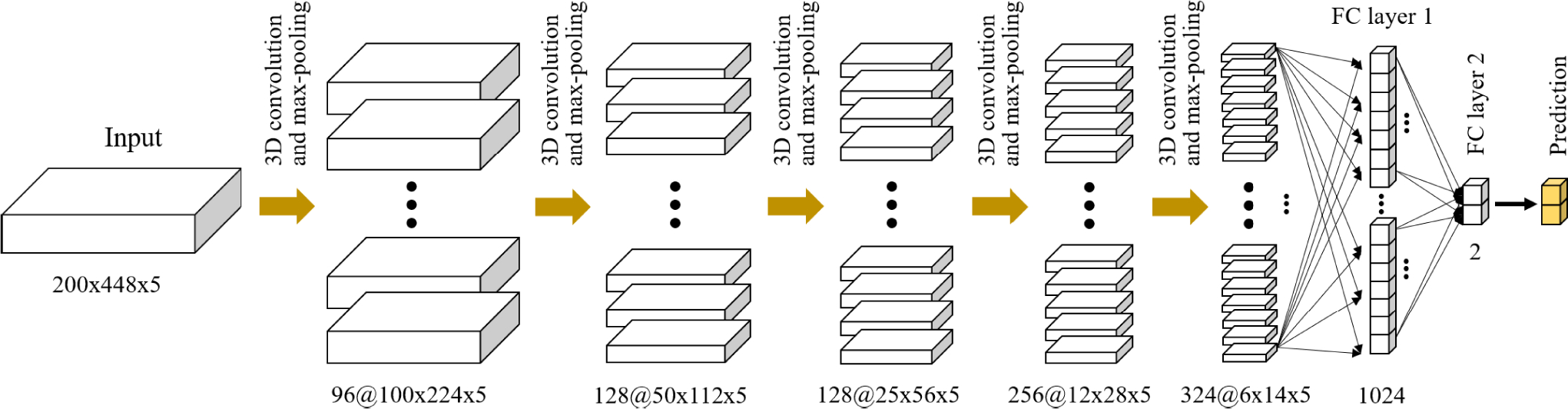
3D CNN architecture for detecting major calcification lesions. The network is composed of five convolutional, five maximum pooling, and two fully connected layers. Each convolutional layer has the same kernel size (3 × 5 × 5) and varying numbers (96, 128, 256, and 324) with a stride of 1 × 1 × 1 pixels. The convolutional process consists of convolutional, batch normalization, and rectified linear unit layers. The kernel size for maximum pooling is set to 2 × 2 × 1. The input is the preprocessed IVOCT volume (200 × 448 × 5), and the output is either calcification or other classes.

To determine the major calcification lesions from the entire pullback, we created a 3D CNN which considered 8,231 IVOCT frames. The network consisted of five convolutional, five max-pooling, and two fully connected layers (Fig. 2). The 3D CNN model was trained to classify each frame as “calcification” or “other”. Each convolutional process consisted of convolutional, batch normalization, and rectified linear unit layers. We used a varying number of filters (96, 128, 256, and 324) and the same filter size of (3×5×5) with a stride of 1 × 1 × 1 pixels. Batch normalization and ReLU layers followed by 3D convolution were used to accelerate the training process. The max-pooling layer, with a pool size of 2×2×1 pixels, was then implemented to reduce dimensionality and prevent overfitting. The two fully connected layers were followed by pairs of convolutional and pooling layers. The first layer included 1,024 outputs with a ReLU activation function and dropout layer. The second layer had two outputs (“calcification” or “other”) with Softmax activation. The *r* (width) paddings were set to zero, as there is no meaningful tissue information. Parametric values were used for the *θ* paddings (height). The top padding (*θ*_*t*_) was obtained from the last A-line of the previous frame; the bottom padding (*θ*_*b*_) was obtained from the top A-line of the next frame. For the first image frame, the last A-line was set to *θ*_*t*_, and the *θ*_*b*_ of the last frame was obtained from its first A-line.

### C. POST-OPTIMIZATION FOR BETTER LOCALIZATION OF THE MAJOR CALCIFICATION LESIONS

**FIGURE 3.**
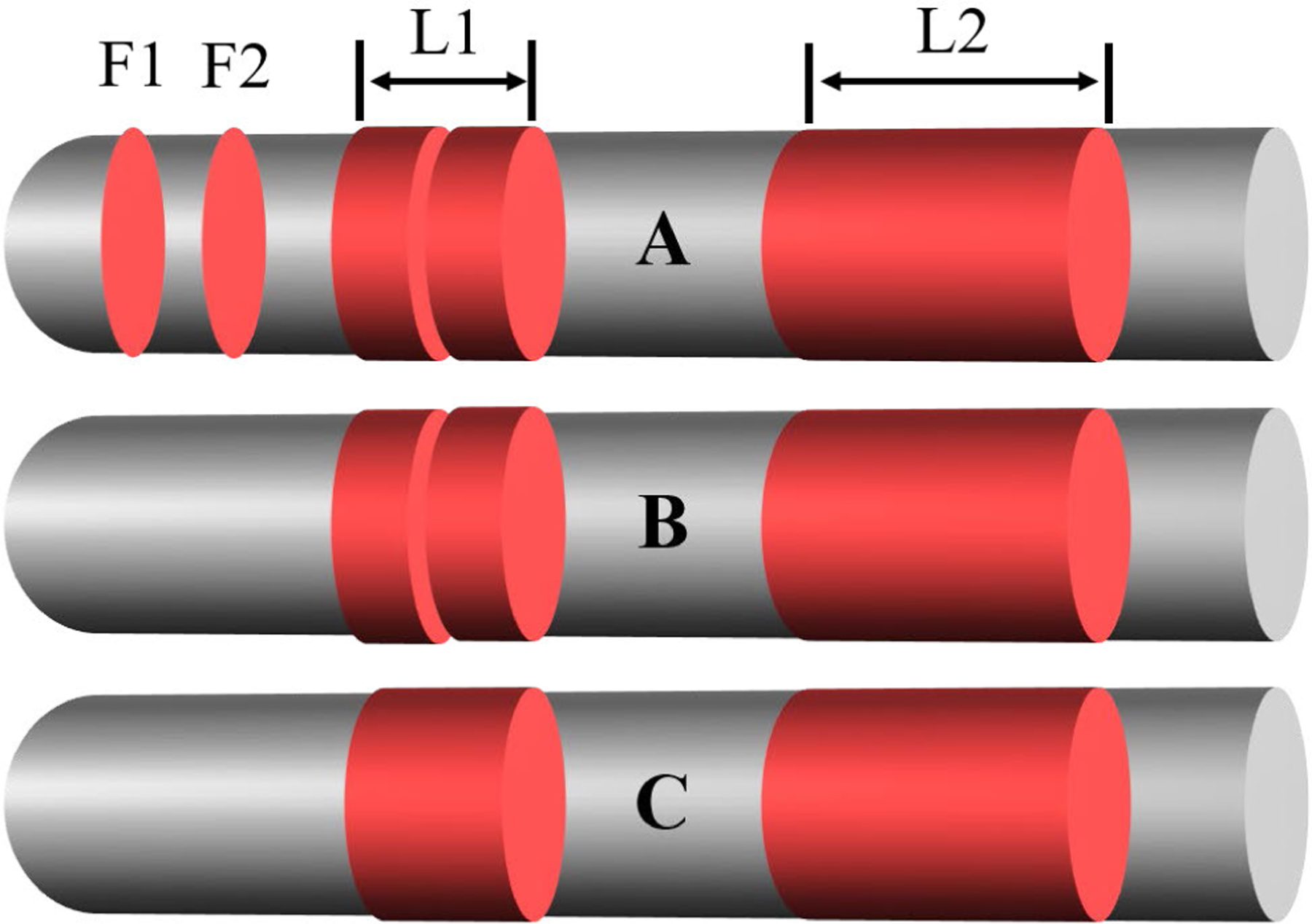
Detection of major calcification lesions (L1 and L2) using 1D morphological operations on a graph. (A) Initial detection result obtained using the 3D CNN. (B) Result after the opening operation. (C) Final result after the closing operation. Gray indicates the entire pullback and red indicates the frames with calcification. The initial classification shown in (A) includes isolated calcification frames (F1 and F2) and missing frames between two areas of calcification (L1). The isolated frames are removed with the opening operation in (B), and the missing frame is merged with the surrounding areas using the closing operation in (C).

The network occasionally produces a few isolated calcification frames or missing frames between calcifications. Isolated calcification-positive frames were ignored, as they are either errors or clinically unimportant in stenting. We used morphological opening and closing operations with a “flat” structuring element of size 5 to remove isolated predictions (Fig. 3). Opening removed isolated calcification frames, while closing filled in the missing frames.

### D. SEGMENTATION OF CALCIFIED PLAQUES USING SEGNET DEEP LEARNING MODEL (STEP 2)

**FIGURE 4.**
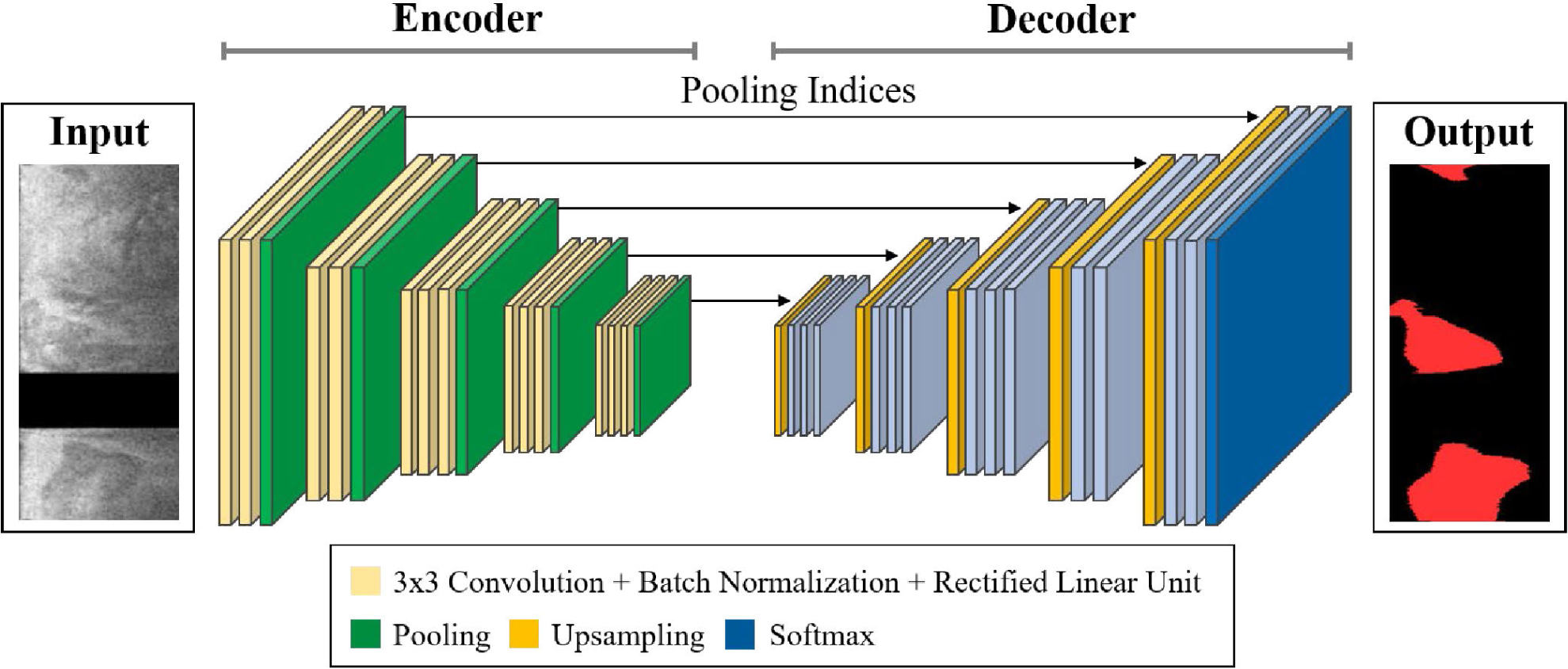
Illustration of SegNet architecture [16] for calcium segmentation. The encoder is composed of a 3 × 3 convolution, batch normalization, and rectified linear unit layers. The decoder upsamples the low-resolution feature map using the transferred pooling indices from the counterpart encoder. The final output of decoder is fed to the Softmax activation to produce a pixel-wise classification map. The input is the preprocessed image selected by the classification model (step 1), and the output is predicted label. The sizes of input and output images are the same (200 × 448 pixels). In the input image, the black strip indicates the removed guidewire shadow.

After determining major calcifications, the selected images were segmented using the SegNet deep learning model [16]. We chose the SegNet as our backbone network, since this model showed better segmentation performance than other conventional CNN models such as Deeplab v3+ in our preliminary study [25]. The SegNet has fully convolutional encoder and decoder networks followed by a final pixel-wise classification layer (Fig. 4). The encoder network uses the general architecture of CNN, which corresponds to the VGG-16 network [35], and removes the fully connected layer to extract important image features. Each encoder comprises a 3×3 convolution, batch normalization, rectified linear unit (ReLU), and 2 × 2 max pooling structure. A ReLU layer was employed to introduce non-linearity. A max-pooling operation with a non-overlapping stride of 2 produced subsampled feature maps between each encoding step. During the 2 × 2 max pooling, the corresponding max-pooling indices (locations) were stored. After all the encoding steps, a low-resolution feature map was obtained, and the feature map was upsampled in the decoder network. For the decoder, a 2×2 max unpooling followed by a convolution was applied to restore the location information encoded at the corresponding encoder layer. The restored feature map was fed to the final classification layer including a 1 × 1 convolution with Softmax activation to produce class probabilities for each pixel. Implementation details are described in Section 3.3.

### E. CLASSIFICATION NOISE REDUCTION USING FULLY CONNECTED CRF AND FRAME INTERPOLATION

CRF is a probabilistic graphical model that constructs the conditional probability of a set of latent variables to each pixel classification [36]. CRF creates a new label with more relevant spatial characteristics of surrounding pixels based on the probability scores generated by the classifier. We implemented a fully connected CRF after our two-step deep learning segmentation. In a fully connected CRF, the pairwise edge potentials are defined by a linear combination of Gaussian kernels and have the following form between any pairs of pixels:

(1) $$\psi_{p}\left(z_{i},z_{j}\right)=\mu \left(z_{i},z_{j}\right)\sum_{q=1}^{Q}\omega^{\left(q\right)}k^{\left(q\right)}\left(f_{i},f_{j}\right)$$

where *k*^(*q*)^ is a Gaussian kernel, the vectors *f*_*i*_ and *f*_*j*_ are feature vectors, *ω*^(*q*)^ are linear combination weights, and *μ* is the Pott’s label compatibility function. A linear combination of Gaussian kernel enables efficient inference with mean field approximation after the message passing step, which can be expressed as a convolution with a Gaussian kernel in an arbitrary feature space. In this implementation, there were three free parameters to be optimized (i.e., the size of the smoothness kernel, weight of the smoothness kernel, and the number of iterations). All were optimized in an ad hoc fashion. The sizes of the smoothness kernels in (*r*, *θ*) domain were set to 1.2 and 1.1, respectively, the weight of the smoothness kernel was set to 0.5, and the number of iterations was set to 10. Detailed descriptions and equations have been described previously [25], [36].

Additionally, we observed that the trained network occasionally produced a “missing frame” with no segmentation in our dataset, even though the adjacent frames were heavily calcified and segmented well. These frames most likely have calcification because plaques are spatially distributed in the vessel wall. In this case, we created intermediate calcification frames by interpolating the adjacent frames and replaced the missing frames.

## III. EXPERIMENTAL METHODS

### A. DATA ACQUISITION

The database used in this study included *in vivo* clinical and *ex vivo* cadaveric IVOCT images. IVOCT images were collected with a frequency-domain ILUMIEN OCT system (St. Jude Medical Inc., St. Paul, Minnesota, USA), which has a tunable laser light source sweeping from 1,250 to 1,360 *nm* at a frame rate of 180 *fps*. The IVOCT catheter was advanced over a conventional guidewire until reaching the segment of interest, and the catheter position was confirmed using X-ray angiography. Imaging pullback was then performed with a pullback speed of 36 *mm/s* and axial resolution of 20 *μm*. The clinical dataset was acquired from 68 patients having 68 entire pullbacks. Exclusion criteria were image frames with poor quality due to luminal blood, unclear lumen, artifact, or reverberation. A total of 8,231 frames across 68 patients were utilized for train/test the networks. For the *ex vivo* cadaveric IVOCT dataset, the two repetitive pullbacks were performed on each of the four cadaver human coronary arteries. Cadaveric arteries included a total of 4,320 frames (2 pullbacks × 4 arteries × 540 frames). The original size of each frame was 968 × 448 pixels in the (*r*, *θ*) domain. The *in vivo* clinical dataset was used to optimize the hyperparameters of the classifier using five-fold cross validation, and the *ex vivo* cadaveric dataset was used for further evaluation of our two-step deep learning approach. This retrospective study was approved by the Institutional Review Board of University Hospitals Cleveland Medical Center (Cleveland, OH, USA).

### B. GROUND TRUTH LABELING

For ground truth labeling, both the clinical and cadaveric raw IVOCT images were Log compressed and converted to Cartesian (*x*,*y*) domain. Clinical images were manually labeled by two expert cardiologists from the Cardiovascular Imaging Core Laboratory, Harrington Heart and Vascular Institute, University Hospitals Cleveland Medical Center (Cleveland, OH, USA), according to consensus standards in [5]. Calcified plaque was determined in heterogeneous signal-poor regions with sharply delineated borders. Residual pixels that did not meet the standard criteria were classified as “other.” Cadaveric IVOCT images were automatically labeled using the previously developed plaque characterization method [25]. Each annotated image was then reviewed and edited by two cardiologists.

### C. NETWORK TRAINING

We used different datasets to train the classification (Step 1) and segmentation (Step 2) networks. For the first step, we used all IVOCT images including “other” and “calcification” classes. Only the calcification frames (4,335) across 47 lesions manually labeled by cardiologists were used for the second step. Classification and segmentation networks were optimized using the adaptive moment estimation (ADAM) optimizer [41], with the initial learning rate, drop factor, and drop period empirically set to 0.001, 0.2, and 5, respectively. The SegNet deep learning model consisted of 26 convolutional, 5 max-pooling, and 5 maxunpooling layers. The initial learning parameters of each encoding layer were determined using the pre-trained deep learning networks (VGG-16 [35] and VGG-19 [35]), and results were compared to find better initialization. We also tested three different loss functions (weighted cross-entropy (WCE) [42], Dice [43], and Tversky [44]) over the Softmax outputs. We fine-tuned weights of each layer at a time starting from the last layer and changed the learning rates of the next layers. In order to prevent over-fitting during the training, the network was trained for a maximum of 50 epochs or until performance on the validation dataset stopped improving over 5 consecutive epochs, whichever occurred first. Since our dataset was imbalanced, we computed the class weight for each class as the inversed median frequency of class proportions, resulting in larger class data having a smaller weight in the loss function and smaller class data to have a larger weight in the loss function. To prevent potential edge effects, we used parametric paddings for all four directions based on the sequential frame information as described in Section 2.2. The receptive field of our SegNet model was 211 pixels, with one pixel padded for each convolution step. All image processing was done using MATLAB (R2018b, Math-Works, Inc.) on a NVIDIA GeForce TITAN RTX GPU (64 GB RAM).

### D. PERFORMANCE EVALUATION

We carried out a five-fold cross validation to evaluate the classification performance of our two-step deep learning approach. A total of 68 pullbacks were divided into 5 independent sub-groups and assigned for training (80%), validation (10%), and testing (10%). Each sub-group was held out for testing while the rest were used for training/validation. Thus, each sub-group was assigned to the test set exactly once to avoid evaluation variance.

Network performance was quantitatively evaluated using traditional metrics as below:

(2) $$\text{Sensitivity}=\frac{TP}{TP+FN}$$

(3) $$\text{Specificity}=\frac{TN}{TN+FP}$$

(4) $$\text{Pr}\text{ ecision}=\frac{TP}{TP+FP}$$

(5) $$F1\;\text{Score}=\frac{2TP}{2TP+FP+FN}$$

where *TP* is the number of true positives, *TN* is the number of true negatives, *FP* is the number of false positives, and *FN* is the number of false negatives. We reported the mean and standard deviation of all metrics over the five folds.

Using the *in vivo* clinical dataset, we compared our two-step approach with the one-step and two-step′ approaches. The one-step approach has only the SegNet model trained on the entire data set without the frame classification step, whereas the two-step′ approach has the same structure as the two-step approach, but SegNet model was trained on the entire data set. To analyze how the training sample size affects the prediction, we evaluated the segmentation performance on the *ex vivo* held-out dataset for varying numbers of training samples, reducing the training sample size from 100% to 10% with 10% intervals. Additionally, we performed a reproducibility test using the *ex vivo* cadaveric dataset by measuring clinically relevant calcium attributes including maximum angle, mean thickness, mean depth, and calcium score [45]. For this purpose, we acquired the initial and repetitive pullbacks from the same lesions at different time points. When testing on the held-out dataset, the network was trained on the entire set of clinical IVOCT images.

## IV. RESULTS

We compared the performance of our 3D CNN model to various 2D models for identifying major calcification lesions. Table 1 compares the mean metrics (e.g., sensitivity, specificity, and F1 score) over the five-folds between the 2D and 3D CNN models. For statistical analysis, the Wilcoxon signed-rank test was performed. GoogLeNet had the lowest sensitivity (87.6±3.5%) and F1 score (0.851±0.068) among all CNN models. The 3D CNN model had the highest sensitivity (97.7 ± 2.4%, p < 0.05) and F1 score (0.922 ± 0.021, p < 0.05) compared to all of the 2D models. 3D CNN successfully detected the major calcification lesions from all IVOCT pullbacks.

**FIGURE 5.**
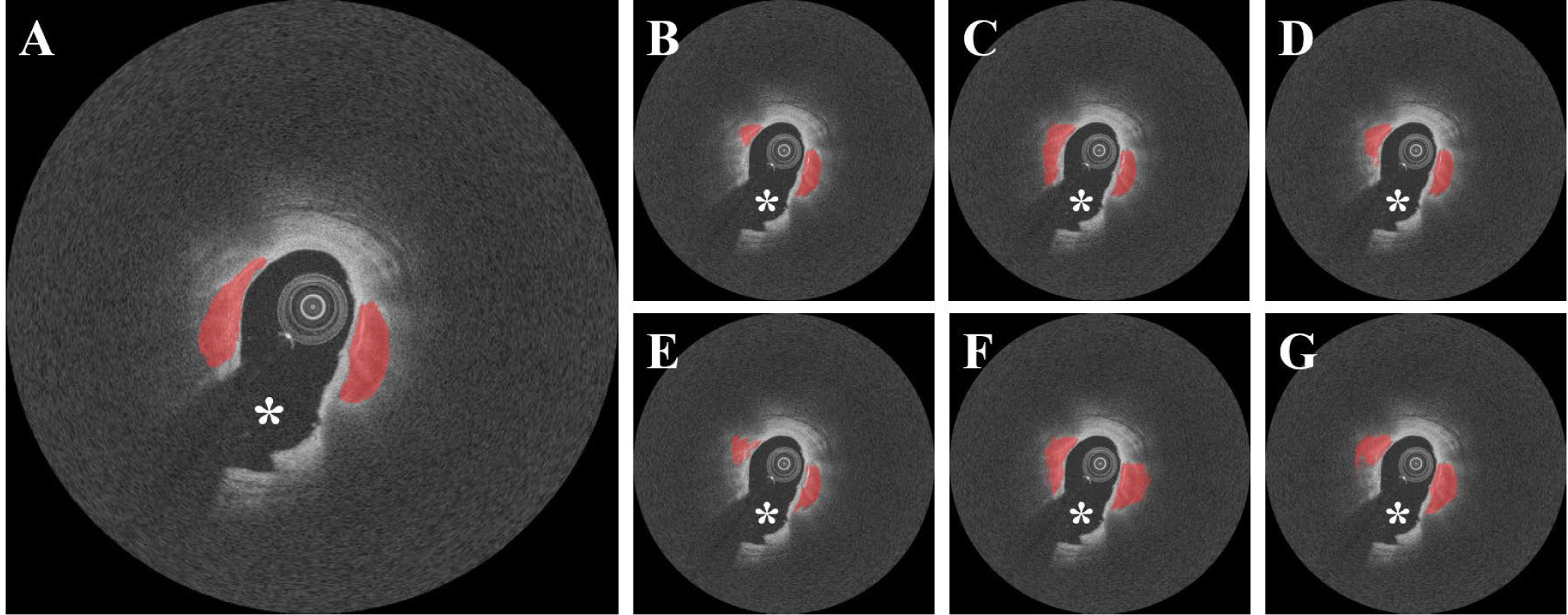
Segmentation results for different deep learning models and loss functions without CRF noise cleaning. (A) Ground truth and results obtained using (B) VGG-16 with WCE, (C) VGG-16 with Tversky, (D) VGG-16 with DICE, (E) VGG-19 with WCE, (F) VGG-19 with Tversky, and (G) VGG-19 with DICE. VGG-16 with the Tversky loss function (C) provided the best segmentation results in terms of F1 score (0.781) and sensitivity (86.2%) among all conditions. The red is the calcified plaque. The white asterisk (*) indicates the guidewire shadow.

We next examined the effect of different combinations of networks and loss functions in the SegNet model on our segmentation classification results. Fig. 5 depicts the calcium segmentations obtained using different combinations of networks and loss functions. The WCE loss function tended to underestimate the calcified plaques, whereas the Tversky and Dice functions estimated the calcified plaques with relative accuracy. We evaluated segmentation with each combination before CRF post-processing to prevent potential misinterpretation. The overall performance of VGG-16 was significantly better than that of VGG-19 (p < 0.05), regardless of the loss function used. The Tversky loss function had a significantly better F1 score (0.781±0.02) and sensitivity (86.2±2.0%) than both WCE (F1 score 0.732 ± 0.046; sensitivity 78.9 ± 5.6%) and Dice (F1 score 0.749 ± 0.021; sensitivity 75.4±7.4%), respectively (p < 0.05). The Tversky function also produced the lowest number of false predictions over all folds. Therefore, we selected the VGG-16 and Tversky loss function as the best training model, and used it to perform all further analyses. We did not see a significant difference in calcium segmentation with the CRF method, which was used for visual improvement.

**FIGURE 6.**
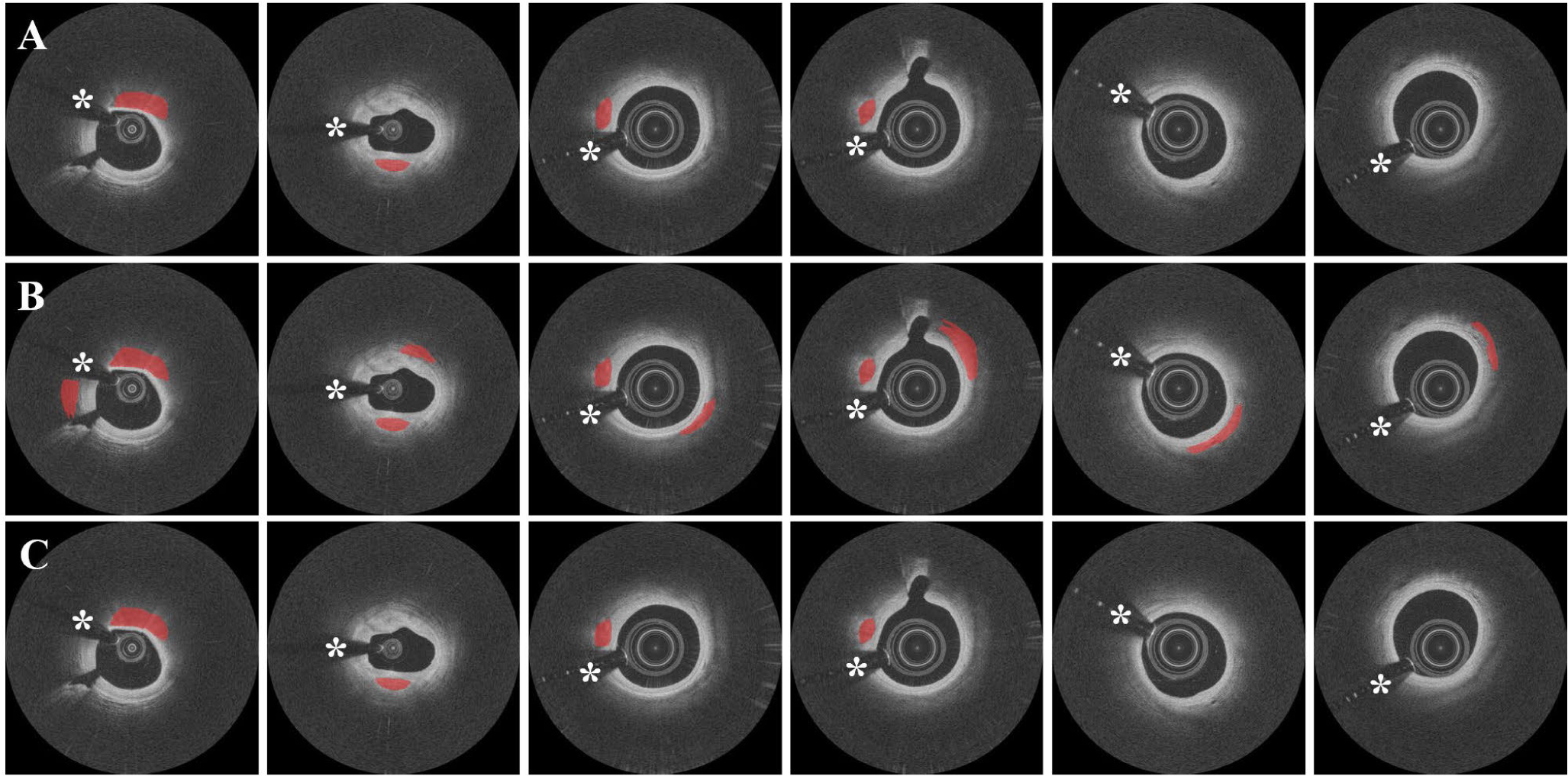
Representative examples of segmentation results for one-step (SegNet trained on all frames), two-step′ (3D CNN + SegNet trained on all frames), and two-step (3D CNN + SegNet trained on calcification frames) approaches. (A) Ground truth images. (B) Results obtained using the one-step and two-step′ approaches. (C) Results obtained using the two-step approach. The one-step and two-step′ approaches misclassified adjacent normal tissues as calcified plaques (columns 1–4) and produced isolated calcification frames (columns 5–6). These misclassifications were not seen with the two-step approach. Red indicates calcified plaque, white asterisk (*) indicates the guidewire shadow.

**FIGURE 7.**
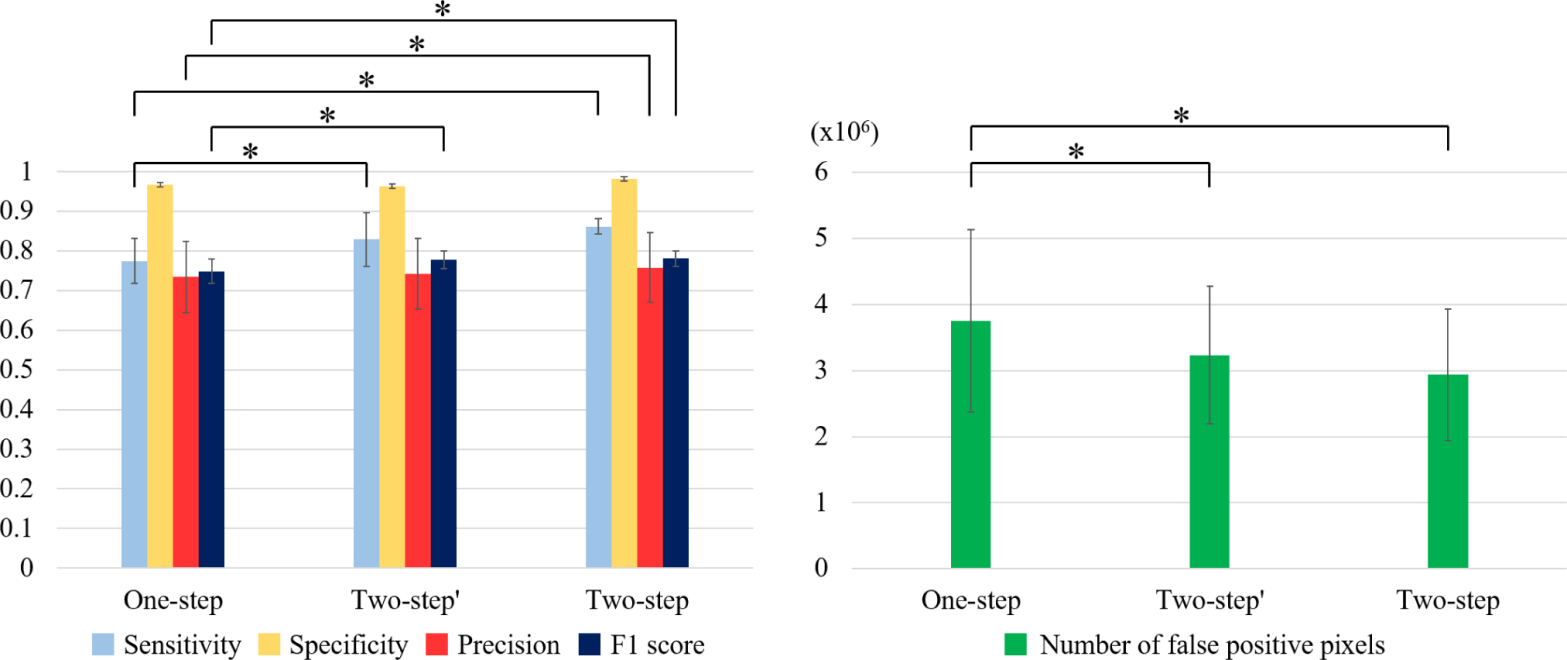
Sensitivity, specificity, and F1 score over five-folds between the one-step (SegNet trained on all frames), two-step′ (3D CNN + SegNet trained on all frames), and two-step (3D CNN + SegNet trained on calcification frames) approaches. (A) The two-step approach showed better segmentation performance compared to the one-step approach, with significantly higher sensitivity, F1 score, and precision (p < 0.05). The standard deviation of sensitivity decreased by more than 60% (from 5.6% to 2.0%). Specificity was slightly better with the two-step approach (96.7 ± 1.1% vs. 98.2 ± 4.0%) (p > 0.05). Additionally, the two-step approach resulted in an improvement in metrics as compared to the two-step′ approach, but the difference was not statistically significant. The two-step′ approach had significantly better sensitivity and F1 score compared to the one-step approach. (B) With the two-step approach, the number of false positive pixels was 22% (3,753,042 vs. 2,936,631) and 9% (3,234,790 vs. 2,936,631) lower, and the standard deviation was 28% (1,380,561 vs. 996,718) and 4% (1,041,704 vs. 996,718) lower, as compared to the one-step and two-step′ approaches, respectively. *p < 0.05.

**FIGURE 8.**
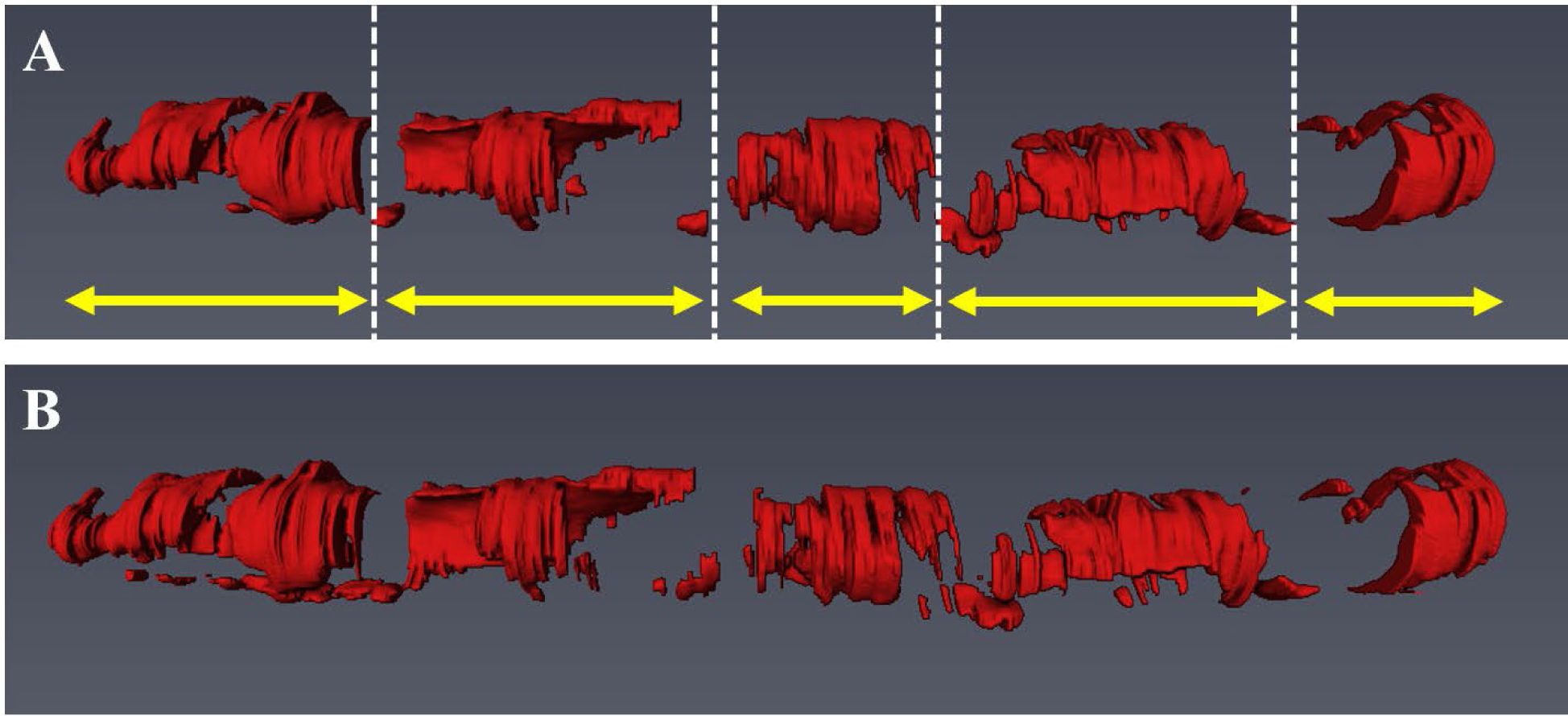
3D visualization of automated detection and segmentation results for major calcification lesions in the entire pullback. (A) Ground truth. (B) Prediction results. Red indicates calcified plaque, and yellow arrows indicate the 5 major calcification lesions. Our two-step deep learning method efficiently detected the major calcification lesions and accurately segmented the calcifications.

We compared the performance of our two-step deep learning approach to the one-step (SegNet trained on all frames) and two-step′ (3D CNN + SegNet trained on all frames) approaches. As shown in Fig. 6, although both one- step and two-step′ networks were able to segment the calcified plaques, they often misclassified the adjacent normal tissues as calcified plaques in the same frames. In contrast, the two-step method did not misclassify frames and had significantly improved sensitivity (77.5±5.6% vs. 86.2±2.0%), precision (73.5±9.0% vs. 75.8±8.8%), and F1 score (0.749±0.03 vs. 0.781±0.02) compared to the one-step method (p < 0.05, Fig. 7). Additionally, the two-step method gave improved metrics as compared to two-step′ method, but the difference was not statistically different. Fig. 8 is a 3D visualization of major calcification and plaque segmentation results obtained using manual annotation vs. our fully automated two-step approach.

**FIGURE 9.**
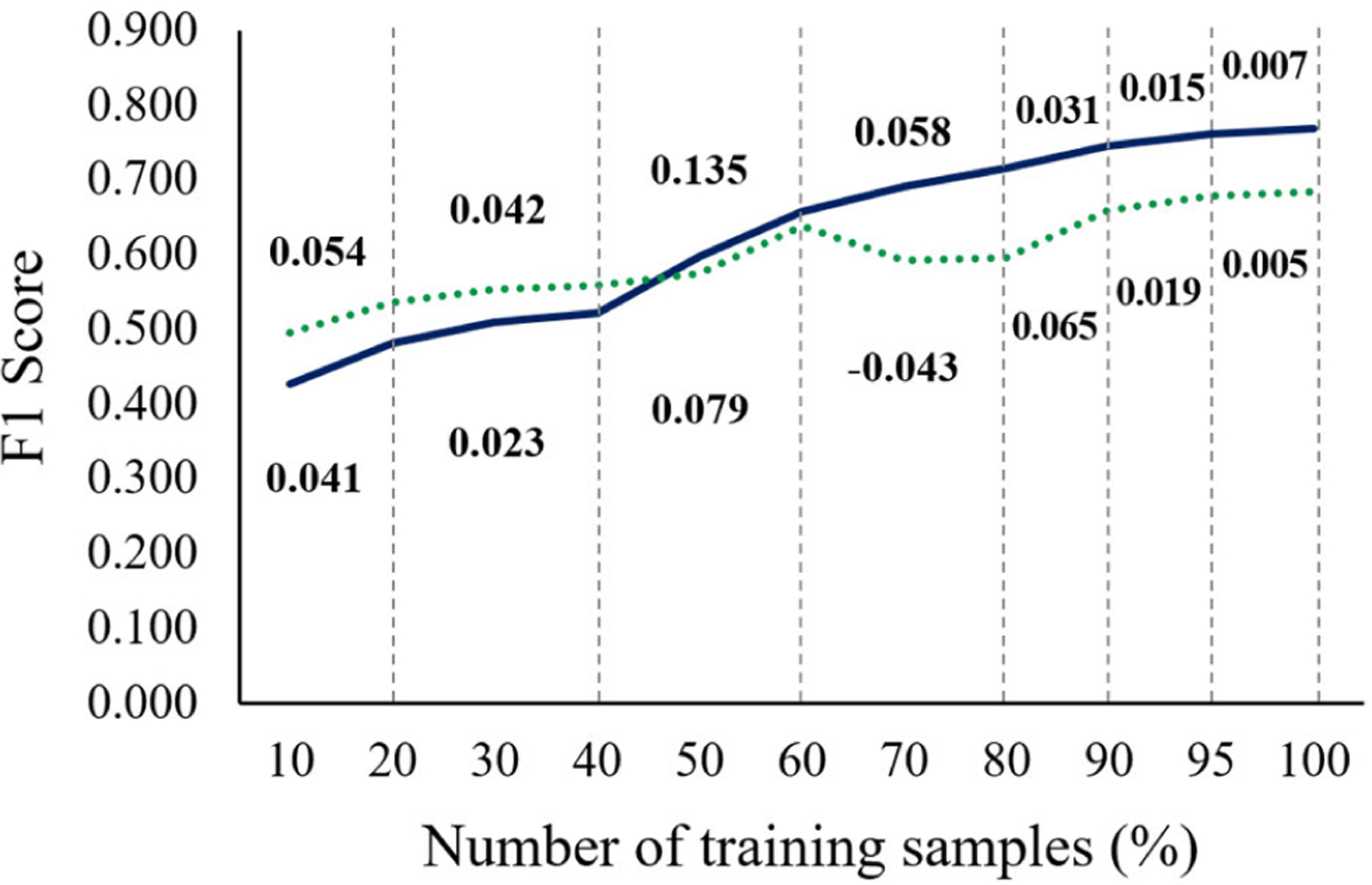
Performance curves of F1 scores for varying numbers of training samples using the one-step (dotted green) and two-step (solid blue) approaches. Ex vivo cadaveric images (4,320 frames) were utilized for the held-out test. Segmentation performance of both approaches continuously improved and reached a plateau after using 90% of the training dataset. The one-step approach required a larger dataset for training, with significantly lower F1 scores compared to the two-step approach after using 80% of the training dataset (p<0.05). The values above and below the graph are the F1 score increments for the two-step and one-step approaches.

We found that our training sample size was reasonable for identifying calcified plaques in IVOCT images. Fig. 9 shows the performance curves for varying numbers of training samples using the one-step and two-step approaches on the *ex vivo* cadaveric held-out test set. For both methods, F1 scores continuously improved as the training sample size increased and then reached a plateau, with very small changes (< 0.015) after using 90% (3,900 frames) of the entire sample set. Thus, at least 3,900 images are needed to obtain stable and standardized results. The one-step approach required a larger dataset and training time, with significantly inferior segmentation performance (p < 0.05) compared to the two-step approach.

**FIGURE 10.**
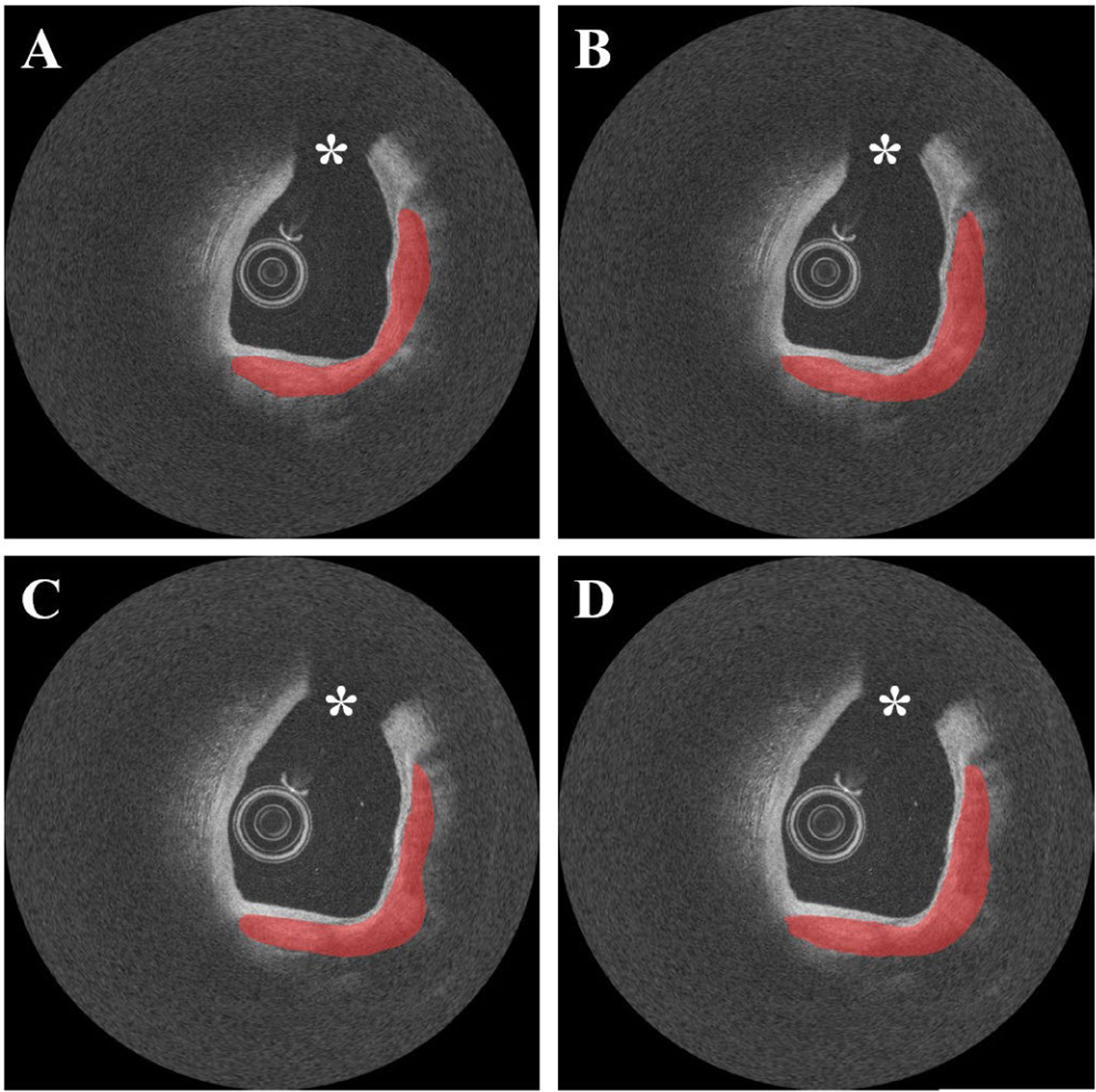
Reproducibility test results on the repeat cadaveric IVOCT pullbacks showing heavily calcified plaques. (A) Ground truth of pullback 1. (B) Prediction of pullback 1. (C) Ground truth of pullback 2. (D) Prediction of pullback 2. Although the cadaveric images have somewhat different intensity profiles, our two-step method produced very similar results on the repeat pullbacks. During the image acquisition, the catheter was placed in the same location. For better visualization, the pullbacks were manually co-registered. Red indicates the calcified plaque, white asterisk (*) indicates the guidewire shadow.

Our method showed excellent reproducibility on the *ex vivo* cadaveric dataset. As shown in Fig. 10, our method produced visually similar results on repeat IVOCT pullbacks on the same calcified plaque lesion. In addition, our method had very small differences in maximum angle (0.8–11.4°), mean thickness (0.014–0.030 *mm*), and mean depth (0.002–0.067 *mm*), and identical calcium scores, on the repeat pullbacks (Table 2).

## V. DISCUSSION

The 3D CNN model had significantly better performance for detecting major calcification lesions compared to the 2D networks. Particularly, sensitivity and F1 score were up to 12% and 8% higher, respectively, for the 3D CNN model. This is likely because 2D networks do not consider the spatial distributions of plaque, though they have deeper network depth and more trainable parameters. Therefore, 2D networks are more likely to produce the isolated false positives (or false negatives) even though the adjacent frames are negative (or positive). The 3D CNN model used in this study is simple but is able to significantly reduce the likelihood of false predictions by taking into account the surrounding frames. Classification performance could be improved further with deeper 3D CNN models.

Step 1 (classification) had more effect on the improvement of segmentation performance than step 2 (segmentation). In our experience with plaque segmentation in IVOCT images, most classification errors occur in mixed tissues where lipidous and calcified tissues coexist. Our approach allows the network to only learn the distinct characteristics of major calcifications by excluding the frames that might cause confusion during training. This key feature of our method permits a robust discrimination between calcified plaques and surrounding tissues. One disadvantage of the one-step segmentation is that it often leads to a greater number of false predictions, such as small islands in the disconnected frame depicted in Fig. 6B. These false predictions do not involve major calcifications and thus are not likely to be clinically important or change treatment decision making. By adding the identification step, we are able to improve segmentation performance by first determining the major calcification lesions in the entire pullback, thereby minimizing the likelihood of misclassifying non-plaque pixels.

The combination of the VGG-16 model and Tversky loss function showed the best segmentation performance. This result is surprising because VGG-19 has more weight layers (19) and trainable parameters (144M) than VGG-16 (16 layers and 138 M parameters). Our findings are likely due to differences in image characteristics (e.g., input size and noise level) rather than differences in the network architecture. Similarly, we expected that the Dice loss function would show the best performance in our dataset (which has class imbalance) because the goal of the Dice loss function is to maximize the metrics. Although WCE has easily differentiable properties, it is not recommended for correcting the imbalance problem. We suggest that the Tversky loss function performed better (or comparably) in terms of sensitivity and F1 score in our dataset because the indices are a generalization of the Dice coefficient, though the image characteristics may still factor into performance.

We found that our training sample size was reasonable for calcium segmentation in IVOCT images. It is crucial to use a large number of training samples for achieving a robust and generalized classification performance, though it is not always clear how much data is needed for training. The amount of training data depends on the difficulty level of the problem. For example, only a few training samples are required for classifying black from white images, while at least 1,000 samples per class are necessary to solve the ImageNet problem [46]. Cho *et al.* [47] created a learning curve for different training sample sizes to investigate how much data is required to achieve the desired accuracy in computed tomography images. They reported that a training dataset per class of 4,092 was needed for their deep learning classifier. Similarly, we found that a minimum training sample size of 3,900 is required to stabilize the segmentation performance in IVOCT images (Fig. 9). Our results may be useful for setting a standard for deep learning applications in IVOCT image analysis.

Our method showed an excellent reproducibility of detecting calcified plaques. For our reproducibility test, we utilized repeat *ex vivo* IVOCT pullbacks acquired from heavily calcified cadaveric coronary arteries rather than the clinical dataset. Although cadaveric arteries might have different tissue properties than living tissues, our method produced similar and robust classification results for all cadaveric IVOCT pullbacks (Fig. 10 and Table 2). Particularly, our method was useful regardless of image type as the attribute differences of each cadaveric artery were small.

Our fully automated method could greatly improve the efficiency of IVOCT image analysis by eliminating the need for manual annotation. Manual analysis of each pullback typically takes around 0.5–2 hours depending on the cardiologist’s experience. On our computer system with non-optimized code, automated analysis takes around 0.3 sec per frame (pre-processing: 0.05s, candidate plaque identification: 0.02 sec, plaque characterization: 0.02 sec, and postprocessing: 0.2 sec) using a Matlab implementation. Our method is currently used for offline analysis of *in vivo* and *ex vivo* IVOCT pullbacks. With faster implementation and algorithm optimization, its application in the clinic would allow for real-time treatment planning.

This study has two main limitations. First, our dataset may include mislabeled ground truth. Although two expert cardiologists annotated the images, it is often difficult to identify the outer boundary of calcified plaque when they are mixed with lipidous plaque due to the quick drop-off in IVOCT signal. Second, there is a possibility that a 3D deep learning model could provide better results than the SegNet model used in this study, since it takes into account the spatial information of plaques.

## VI. CONCLUSION

We developed a fully automated calcium segmentation method in IVOCT images using a two-step deep learning approach. We found that training the network with only the major calcification lesions significantly improves segmentation results compared to one-step approaches that train with the entire dataset. Additionally, our method had excellent reproducibility. We predict that this method will have applications in both research and real-time image analysis and treatment planning.

## Figures and Tables

**TABLE 1.** Classification Results for the Major Calcification Lesions.

Networks	Sensitivity (%)	Specificity (%)	FI score
GoogLeNet [37]	87.6±3.5	81.5±8.7	0.851±0.068
ResNet-101 [21]	88.8±2.0	87.9±5.0	0.888±0.032
DenseNet-201 [22]	92.5±2.6	88.7±4.0	0.910±0.023
Inception-v3 [38]	91.9±3.0	87.9±4.1	0.903±0.029
Xception [39]	92.5±3.0	89.0±5.2	0.911±0.030
Inception-ResNet-v2 [40]	91.6±2.8	86.6±5.4	0.895±0.034
3D CNN	97.7±2.4	87.7±2.1	0.922±0.021

**TABLE 2.** Calcification Attributes from the Reproducibility Test on the Cadaveric Held-out Test Set.

Reproducibility		Maximum Angle (°)	Mean Thickness (mm)	Mean Depth (mm)	Calcium Score
Artery 1	Initial Pullback	134.2±77.3	0.477±0.184	0.150±0.140	4
	Repetitive Pullback	138.2±78.5	0.498±0.175	0.132±0.099	4
Artery 2	Initial Pullback	68.5±40.2	0.367±0.166	0.207±0.135	1
	Repetitive pullback	79.8±41.2	0.398±0.159	0.274±0.212	1
Artery 3	Initial pullback	109.9±40.2	0.588±0.142	0.129±0.060	4
	Repetitive pullback	111.1±40.4	0.611±0.140	0.127±0.060	4
Artery 4	Initial pullback	83.9±46.0	0.477±0.217	0.159±0.121	4
	Repetitive pullback	81.8±46.5	0.491±0.222	0.178±0.136	4
